# Victimization experiences and the stabilization of victim sensitivity

**DOI:** 10.3389/fpsyg.2015.00439

**Published:** 2015-04-14

**Authors:** Mario Gollwitzer, Philipp Süssenbach, Marianne Hannuschke

**Affiliations:** Department of Psychology, Philipps-University Marburg, Marburg, Germany

**Keywords:** victim sensitivity, personality development, stabilization, cognitive processes, social justice

## Abstract

People reliably differ in the extent to which they are sensitive to being victimized by others. Importantly, “victim sensitivity” predicts how people behave in social dilemma situations: Victim-sensitive individuals are less likely to trust others and more likely to behave uncooperatively—especially in socially uncertain situations. This pattern can be explained with the sensitivity to mean intentions (SeMI) model, according to which victim sensitivity entails a specific and asymmetric sensitivity to contextual cues that are associated with untrustworthiness. Recent research is largely in line with the model’s prediction, but some issues have remained conceptually unresolved so far. For instance, it is unclear why and how victim sensitivity becomes a stable trait and which developmental and cognitive processes are involved in such stabilization. In the present article, we will discuss the psychological processes that contribute to a stabilization of victim sensitivity within persons, both across the life span (“ontogenetic stabilization”) and across social situations (“actual-genetic stabilization”). Our theoretical framework starts from the assumption that experiences of being exploited threaten a basic need, the need to trust. This need is so fundamental that experiences that threaten it receive a considerable amount of attention and trigger strong affective reactions. Associative learning processes can then explain (a) how certain contextual cues (e.g., facial expressions) become conditioned stimuli that elicit equally strong responses, (b) why these contextual untrustworthiness cues receive much more attention than, for instance, trustworthiness cues, and (c) how these cues shape spontaneous social expectations (regarding other people’s intentions). Finally, avoidance learning can explain why these cognitive processes gradually stabilize and become a trait: the trait which is referred to as victim sensitivity.

## Introduction

Imagine the following situation: your colleague asks you to do a favor for her, such as switching shifts with her because she says she needs to see a doctor. You agree and take her early-morning shift. A couple days later, you learn that your colleague never saw a doctor (and never intended to do so); instead, she needed to sleep in that day because she had been partying the other night. What you probably feel in that very moment is a mixture between anger, moral outrage, disappointment, helplessness, and regret. You trusted your colleague, but your trust was betrayed, and you will most probably decide that you will never trust her again—and maybe you will not even trust any other of your colleagues. The incident has probably made you more sensitive to the fact that other people can exploit your goodwill.

Although such incidents of betrayed trust are certainly aversive to everyone, the extent to which people react emotionally to such an incident and ruminate about the injustice that it implies differs considerably between individuals: some people merely feel a sting of anger which quickly dissolves as time goes by. Others experience a powerful and overwhelming range of negative emotions and ruminate for a long time about the incident and what it says about them. The latter kind of individuals can be referred to as having a strong sensitivity to injustice from the victim’s perspective (or “victim sensitivity”). Victim sensitivity is a personality trait that has originally been developed to measure individual differences in the justice motive (Schmitt et al., 1995; Schmitt, 1996). Later, it has been conceptualized as one of four perspectives from which people can be sensitive toward injustice (the other perspectives are: observers, beneficiary, and perpetrator; cf. Schmitt et al., 2010). Unlike the other perspectives, victim sensitivity has been found to predict suspicious cognitions, social mistrust, egoism, and uncooperativeness (Fetchenhauer and Huang, 2004; Gollwitzer et al., 2005; Gollwitzer and Rothmund, 2011). According to a model that aims at explaining these effects (i.e., the “sensitivity to mean intentions” or SeMI model; cf. Gollwitzer and Rothmund, 2009; Gollwitzer et al., 2013), victim-sensitive individuals can be characterized as harboring a latent fear of being exploited and as being chronically hypersensitive to cues that are associated with untrustworthiness. From this perspective, their antisocial and egoistic behavior can be conceptualized as a defensive reaction to prevent exploitation: victim-sensitive individuals behave uncooperatively toward others because they expect others to behave uncooperatively toward them.

Many empirical findings are in line with that notion: Victim-sensitive individuals are more sensitive to even slight cues of untrustworthiness (Gollwitzer et al., 2009, 2012), even if these cues have only limited prognostic validity for a situation in which one might be exploited (Rothmund et al., 2011, 2015). Victim-sensitive individuals are more likely to behave aggressively (Bondü and Krahé, 2014) and destructively, especially if they sense a risk of being exploited (Schmitt and Mohiyeddini, 1996; Mohiyeddini and Schmitt, 1997; Schmitt and Dörfel, 1999). They make more egoistic choices in social dilemmas (Fetchenhauer and Huang, 2004), and are less willing to help others in need (Gollwitzer et al., 2005), both in interpersonal and in intergroup situations (i.e., when there is a certain danger that the goodwill of one’s ingroup might be exploited by an outgroup; Süssenbach and Gollwitzer, 2015). They are more envious and more jealous (Schmitt et al., 2005), less willing to accept apologies from their partners (Gerlach et al., 2012), and more likely to oppose political reforms because they think that politicians act out of ulterior motives (Agroskin et al., in press).

As any personality trait that deserves this attribute, victim sensitivity remains relatively stable over time: In a representative sample of German adults (mean age: 47.6 years), 60% of the true-score variance in victim sensitivity, measured at three occasions with a time lag of 2 years, can be attributed to a latent trait, whereas only 33% of the true-score variance can be attributed to occasion-specific influences (Schmitt et al., 2005). In line with this finding, several studies have shown that victim sensitivity reliably predicts social behavior in lab experiments even though victim sensitivity was measured weeks or even months before the lab experiment took place (e.g., Gollwitzer and Rothmund, 2011; Gollwitzer et al., 2012). This stability is remarkable, and it demands a psychological explanation. What makes victim sensitivity a stable trait? As we will see, addressing this question requires an elaborate theoretical framework assuming systematic interactions between social experiences, cognitive representations, and learning processes. We will sketch such a theoretical framework in the present article.

The overarching question—how victim sensitivity stabilizes—consists of two facets or sub-questions. A first sub-question concerns the “ontogenetic” development of victim sensitivity: when do individuals begin to become victim-sensitive, and what are the psychological processes that catalyze the emergence and stabilization of victim sensitivity during the life course? Our attempt to provide answers to this question bears on insights from life-span developmental psychology and personality psychology. The second sub-question concerns the “actual-genetic” development of victim sensitivity: how does victim sensitivity stabilize in the course of specific social situations in which justice and trustworthiness are an issue—situations like the one we described at the beginning of this article? How do victim-sensitive individuals perceive and interpret such situations, and how do these perception and interpretation processes contribute to a further stabilization of victim sensitivity? Our attempt to provide answers to this particular question mainly refers to research on associative learning and social cognition. We believe that the General Process Model of Threat and Defense (Jonas et al., 2014) is particularly suitable to explain how victim-sensitive individuals react to cues associated with untrustworthiness in their social worlds.

## When and How Does Victim Sensitivity Begin to Emerge and Stabilize?

The “SeMI” model assumes that victim sensitivity is rooted in a specific cognitive dissonance: the dissonance between a need to trust others and a stable expectation that others are not trustworthy (Gollwitzer and Rothmund, 2009). According to the SeMI model, victim-sensitive individuals would love to live in a world in which other people can be trusted, in which the risk of being exploited is close to 0, and in which cooperation is always likely to pay off for everybody in the end. However, at one or several points in their lives, these individuals have experienced that other people are not as trustworthy and as reliable as they had hoped. We assume that such victimization experiences establish the basis for developing victim sensitivity. More concretely, we hypothesize that if victimization experiences constitute “critical” life events and if these events are coped with in a dysfunctional way, victim sensitivity is likely to increase and stabilize. Victimization experiences can have many different faces. Victimization can mean emotional or even physical abuse, betrayal of trust, or social rejection. All of these different experiences have one thing in common: they thwart a particular need, the need to trust.

### The Need to Trust

The need to trust other people has been conceptualized as one of the five “core social motives” (Fiske, 2009). To trust means to believe in other people’s trustworthiness, that is, in their abilities, their integrity, and—most importantly—their benevolence (cf. Mayer et al., 1995). Trusting others is not only beneficial; it is essential for maintaining relationships and contributing to social groups. Trust helps us master uncertain or novel situations; it is a key component in many social interactions, from bargaining to loving, and it is considered to be at the roots of economic systems, the core of social capital, and the driving machine of democratic societies (Coleman, 1990; Putnam, 2000).

Integrity and benevolence are especially relevant in interdependence situations, that is, when the effect of one’s own behavior on the desirability of different outcomes crucially depends on the behavior of other people (Thibaut and Kelley, 1959; Kelley and Thibaut, 1978). One particular type of interdependence situation is the “social dilemma” (cf. Komorita and Parks, 1995), in which one’s own willingness to cooperate with others or to contribute to a common good might be exploited by others. Typical social dilemmas are the prisoner’s dilemma, the public goods dilemma, or the trust game. The trust game, for instance, consists of two players (cf. Berg et al., 1995). One player, the “truster,” can decide to entrust a certain amount of his or her endowment to the other player. This amount is then multiplied by the experimenter and transferred to the other player (the “trustee”), who can then decide to split the total amount or to keep it all for him-/herself. The principal is: trusting one’s partner can benefit both players, but only if the “trustee” is cooperative. The situation described at the beginning of this article is a typical “trust game” situation: your colleague asks you for a favor, and your willingness to help her might either be exploited (which was the case in this example) or rewarded because you actually helped her in a difficult situation. Trust is the most important predictor of one’s behavior in these kinds of games (e.g., Pruitt and Kimmel, 1977; De Cremer, 1999), and distrust (due to a fear of being exploited) strongly predicts one’s unwillingness to cooperate (Coombs, 1973; Orbell and Dawes, 1981; Kerr, 1983). Given that trust is so immensely functional, both on the interpersonal as well as on the intergroup level, it makes sense to assume that trusting others is something that people are motivated to do in general.

Theories of psychosocial development echo the notion that trust is a basic human motive and that the opportunity to lead a happy, healthy life depends on whether people have developed a general sense of trust in their social worlds. Erikson’s (1950, 1959) theory of life tasks (and their resolution) assumes that the very first task in life is to develop trust in a caregiver. A toddler whose basic needs (such as food, warmth, and closeness) are thwarted is—according to this theory—likely to develop a deep sense of mistrust, anxiety, and insecurity in later life. In a similar vein, attachment theory (Bowlby, 1982, 1988) also focuses strongly on the infant-caregiver bond and highlights the importance of support and caregiving processes for the development of trust and for the quality of intimate relationships in later life. More precisely, attachment theory posits that early parent–child interactions provide the basis for the development of inner working models (Bowlby, 1982) by forming expectations regarding future interactions. Inner working models correspond to mental representations of oneself, of others, and of relationships in general. These representations result in attachment patterns, which can be qualitatively categorized into “secure” vs. “insecure” attachment styles (e.g., anxious/ambivalent, anxious/avoidant, and disorganized; Ainsworth et al., 1978). Notably, “insecure” attachment styles are associated with representations of others as being untrustworthy and of oneself as being incapable (and/or unworthy) of obtaining others’ cooperation.

Taken together, these theories imply that the capability (or the willingness) to trust others as an adult may depend strongly on the kind of experiences people have had in their childhood. However, this does not necessarily mean that generalized expectations regarding other people’s trustworthiness crystallize in early childhood. Empirical findings rather suggest that social trust stabilizes later—especially between early and late adolescence (e.g., Flanagan and Stout, 2010). Thus, adolescence may be considered a critical period in life in which social trust crystallizes and in which people shape their general views about the trustworthiness of other people in accordance with the kind of experiences they had. Additionally, findings from life-span developmental psychology have shown that parental influences on the child’s personality development decrease gradually during late childhood and especially during early adolescence, whereas “extra-familial” influences, such as peers, friends, and especially intimate partners, become increasingly relevant (Caspi, 1998).

### Victimization Experiences

Social experiences are likely to shape the formation of trust and expectations regarding the trustworthiness of others. The question is which kinds of social experiences have the potential to affect these expectations. We assume that expectations concerning other people’s *un*trustworthiness are learned via experiences of victimization (cf. Baumert and Maltese, 2014). These experiences could include *direct* as well as *observed* victimization.

#### Direct Experiences of Victimization

Childhood and adolescence are rife with situations that challenge the notion that our fellow humans’ intentions are universally good and benevolent. In early adolescence, such victimization experiences can include physical or emotional abuse (Björkqvist et al., 2011), (cyber)bulling (König et al., 2010), or unfair treatment by authorities (Pretsch et al., in press). These situations imply violations of fairness standards—standards of distributive fairness (e.g., equality, equity, or need), of procedural fairness (e.g., the opportunity to “voice” one’s opinion), or of interactional fairness (e.g., the right to be treated respectfully). We assume that such violations, especially if they occur repeatedly and if they constitute “critical” life events (see below), contribute to the development and stabilization of victim sensitivity during childhood and adolescence. In addition, experiences of *social rejection*—that is, being excluded from a social relationship or social interaction—are likely to contribute to the development of victim sensitivity as experiences of social rejection can advance generalized negative expectations concerning others’ trustworthiness. Relevant experiences of social rejection include parental rejection, but also peer rejection or indirect bullying (cf. Rivers and Smith, 1994; Ettekal and Ladd, 2015).

#### Observed Experiences of Victimization

Although some degree of directly experienced victimization is probably necessary for the development of victim sensitivity, experiences of victimization that are observed from a third-party perspective are likely to play a role as well. Observing social rejection, interpersonal transgressions, and violations of fairness standards might be just as suited to form generalized negative expectations concerning others’ trustworthiness as actually experiencing them. Drawing on research on vicarious traumatization (McCann and Pearlman, 1990), observed experiences of victimization might be particularly influential under conditions that promote empathy with the victim, for instance, when a family member or one’s best friend is bullied, exploited, or otherwise treated badly. Notably, observed victimization of (significant) others may elicit moral outrage and motivate observers to fight against injustice on behalf of the victim—but these observations may nonetheless make observers more sensitive to victimization. A special instance of observed victimization is witnessing injustice in the media. Media consumption can have sustainable effects on normative beliefs, values, and self- as well as world views (Huesmann and Guerra, 1997; Möller and Krahé, 2009). For example, Rothmund et al. (2015) have recently demonstrated that exposure to violent video games at the age of 14 can contribute to a decrease in interpersonal trust 1 year later. These findings suggest that not only directly experienced, but also indirectly experienced confrontations with violence and untrustworthiness (e.g., in the media) can influence adolescents’ trustworthiness expectations (see also Rothmund et al., 2013).

#### Victimization Experiences as Critical Life Events

Building on research from life-span developmental psychology, certain victimization experiences—both directly experienced and indirectly observed ones—can be considered “critical” life events. Critical life events are specific kinds of stressors that can be differentiated from “normal” life events by several characteristics (see below; Filipp and Aymanns, 2010). Among these are (1) the extent to which the event is informative about oneself (i.e., relevant for one’s self-concept or self-esteem), (2) the extent to which the event interferes with plans and reduces the freedom to act, (3) the unpredictability, and (4) the uncontrollability of the event. The more a victimization experience is self-relevant, goal-obstructing, unpredictable, and uncontrollable, the more likely it will have a strong impact on general beliefs about trustworthiness and the stabilization of those beliefs. Again, not only directly experienced instances of victimization, but also indirectly observed instances of victimization can constitute critical life events that can shape a person’s dispositional untrustworthiness expectations. For instance, learning that one’s best friend had been exploited and cheated upon by his or her partner for years can reduce one’s trust into others—maybe even to the same extent as having suffered exploitation oneself can do.

In addition, individual characteristics, vulnerabilities, and resources (e.g., self-concept aspects, individual norms, sensitivities, interpersonal integration, opportunities for social support, etc.) are relevant for how a person copes with the event. The extent to which a particular victimization experience shapes trustworthiness expectations (and, thus, promotes the stabilization of victim sensitivity) thus depends on characteristics of the event itself *in conjunction with* characteristics of the person.

### Social Information Processing Patterns

One such person characteristic is how people tend to perceive, interpret, and react to social situations. The social information-processing (SIP) model of children’s social adjustment (Crick and Dodge, 1994) assumes that these perceptions, interpretations, and reactions to social events are critically influenced by so-called “data base” information stored in memory. This “data base” consists of general social knowledge structures such as inner working models of relationships (Bowlby, 1982), cognitive schemas, self-concepts, and behavioral scripts (Schank and Abelson, 1977). When confronted with particular social situations, individuals often rely on this social knowledge. Thus, the “data base” critically influences how cues are perceived and interpreted and how people react toward these cues. And, in the sense of a feedback loop, social situations and their outcomes may stabilize and reinforce this social knowledge if the outcomes are consistent with prior expectations.

The notion of a “data base” in the SIP model (Crick and Dodge, 1994) is perfectly compatible with the SeMI model (Gollwitzer and Rothmund, 2009; Gollwitzer et al., 2013). The SeMI model proposes that being confronted with contextual cues associated with untrustworthiness evokes a “suspicious mindset” among victim-sensitive individuals. Past experiences of betrayal, rejection, or unfair treatment (which, according to the SIP model, are stored in a person’s “data base”) thus contribute to a generalized expectation that people are not trustworthy and unreliable, an attributional bias including a heightened availability of hostile interpretations of others’ intentions, and a stabilized behavioral script that favors uncooperativeness in social exchange situations. As we will discuss in Section “How Does Victim Sensitivity Perpetuate Itself Across Social Situations?”, the way victim-sensitive individuals perceive, interpret, and react to social encounters in which untrustworthiness cues are present reinforces their cognitive schemas, and thus, their dispositional victim sensitivity even further.

### Ontogenetic Stabilization Processes

In the previous paragraphs we have discussed which kinds of victimization experiences—in combination with particular personal characteristics—are likely to contribute to the emergence and stabilization of victim sensitivity during childhood and adolescence. We will now discuss the processes that may be useful to explain how victim sensitivity stabilizes “ontogenetically” over time. First, we will discuss self-stabilization and environment stabilization as two important sources of stabilization according to life-span personality psychology (e.g., Lang et al., 2006). Next, we will discuss person-environment transaction processes and their relevance for the stabilization of victim sensitivity.

#### Self- and Environment Stabilization

Personality theories focus mainly on three different sources for stabilization: (1) an increasing self-stabilization, (2) an increasing stabilization due to a more stable environment, and (3) a stabilizing contribution of the genome.^1^ *Self-stabilization* refers to the stabilization of self-relevant knowledge, one’s self-concept, over time (Kagan, 1980). Victim-sensitive individuals might develop a “victim self-concept,” which includes self-related views such as “I am easy prey” or “I am a person who attracts the attention of bullies;” and the stabilization of such a self-concept may, in turn, increase (and stabilize) one’s sensitivity to victimization. *Environment stabilization*, on the other hand, means that social environments become increasingly stable across the life span, which, in turn, also has a stabilizing effect on one’s personality. Self- and environment stabilization processes are not independent of each other; nevertheless, personality → environment effects can be empirically differentiated from environment → personality effects via longitudinal studies (e.g., Asendorpf and Wilpers, 1998). In general, “core” personality traits (such as the “Big Five”) have a stronger effect on the environment than *vice versa*, whereas “surface” personality traits (such as self-worth or loneliness; cf. Asendorpf and van Aken, 2003) are more likely to be shaped by environments. For instance, Asendorpf and van Aken (2003) found that extraversion (a “core” personality trait) predicted changes in social relations (e.g., increased support from peers), but not *vice versa*; changes in global self-worth or loneliness (two “surface” traits), however, were predicted by social relations, but not *vice versa*. Victim sensitivity can be conceptualized as having both “core” and “surface” characteristics. Thus, personality → environment effects *of* victim sensitivity are likely to be as strong as environment → personality effects *on* victim sensitivity.

#### Person-Environment Transactions

Dynamic-interactionistic approaches explain the stabilization of personality by an increasing “fit” between persons and the environments they find themselves in (Caspi, 1998). According to Caspi and Roberts (1999, 2001), this increase in fit is a function of four potential “transactions:” (1) reactive transactions, (2) evocative transactions, (3) selective transactions, and (4) manipulative transactions. We will now discuss these transactions—and their relevance for the stabilization of victim sensitivity in particular—in more detail.

*Reactive transaction* refers to the fact that different individuals react differently to the same objective situation. As the SIP model (Crick and Dodge, 1994) as well as social-cognitive personality theories (e.g., Bandura, 1999; Cervone and Shoda, 1999; Shoda and Mischel, 2000; Fleeson, 2001) suggest, cognitive schemas and behavioral scripts shape how a person perceives, attributes, and reacts to social situations (see also Social Information Processing Patterns). In turn, consistently applying these perceptions, attributions, and reactions also reinforces—and, thus, stabilizes—the schema. Consistently attributing “mean intentions” to others reinforces a person’s victim sensitivity. In other words, schema-congruent information processes imply a *confirmation bias* that stabilizes the schema (Nickerson, 1998).

*Evocative transactions* refer to the processes by which people elicit reactions from others that are consistent with their *a priori* expectations. This stabilizes these expectations. Stated differently, people’s behavioral patterns create a consistency in other people’s reactions toward them; a “self-fulfilling prophecy.” If victim-sensitive individuals perceive and interpret situations against the background of their negative assumptions (others’ untrustworthiness) and react accordingly (e.g., uncooperatively), others may react to this behavior in a similar way (e.g., uncooperatively), which, in turn, confirms the negative beliefs that victim-sensitive individuals have about other people’s untrustworthiness (see also How Does Victim Sensitivity Perpetuate Itself Across Social Situations?).

*Selective transactions* refer to the active selection of environments. Based on their individual preferences, attitudes, and competences, people actively seek out environments that “fit” their personality. For instance, adolescents prefer peers that are similar to themselves; this preference, in turn, stabilizes behavioral dispositions due to social reinforcement (Newcomb et al., 1993; Harris, 1995). Victim-sensitive individuals may thus select friends, partners, colleagues, etc., who are similarly suspicious about others’ intentions as they are. This “confirms” the correctness of their (negative) assumptions and stabilizes them accordingly.

Finally, *manipulative transactions* involve active behaviors that establish environments which are consistent with one’s own individual experiences and behaviors. Victim-sensitive individuals might influence their environment (their friends, colleagues, relatives, and children) to become just as suspicious as they are. By manipulating their environment in this way, victim-sensitive individuals therefore “create” social relationships that are in line with their own expectations, which, in turn, stabilizes their victim sensitivity even further.

According to Caspi (1998; see also Caspi et al., 1989), these four transactions can influence person-environment fit both in a direct and in a more indirect way. The indirect way describes a cumulative effect over a longer period of time. The latter one is also referred to as the principle of “cumulative continuity.” It assumes that the possibility to establish a person-environment fit increases as one gets older. This implies that the stability of personality traits increases as a function of our capacity to select and control the environments we live in.

### Conclusion

**FIGURE 1:**
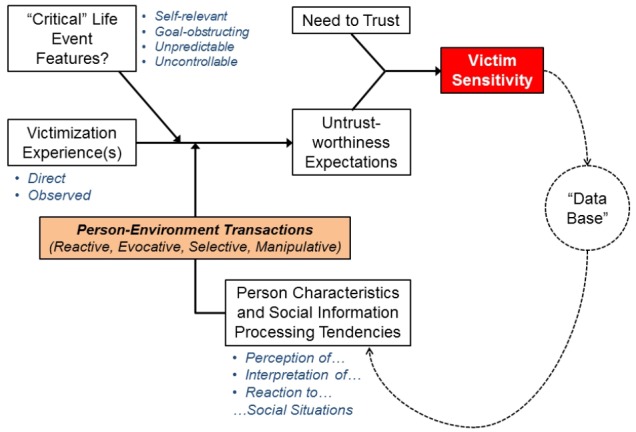
**Theoretical model explaining the “ontogenetic” stabilization of victim sensitivity across the life-course**.

The arguments we discussed and the theories and studies we reviewed so far can be used to describe a model which describes the “ontogenetic” stabilization of victim sensitivity (see Figure 1). We started this discussion by referring to the “need to trust” as a core social motive that is likely to be innate and that requires attention and satisfaction already at very early age. As any other motive, the “need to trust” may differ between individuals, but a certain level of this need can most likely be found in all humans. Nevertheless, people with a strong need to trust may be particularly likely to develop a high sensitivity to victimization later in life.

Given its strong motivational component, people become sensitive to instances in which the need to trust is thwarted. We referred to these instances as victimization experiences. Victimization can be directly experienced or observed from a third-party perspective. More importantly, victimization experiences can constitute “critical” life events if they are (a) self-relevant, (b) goal-obstructing, (c) unpredictable, and (d) uncontrollable. Depending on characteristics of the person (i.e., vulnerabilities, sensitivities, opportunities for social support, etc.) and—especially—on habitual tendencies to perceive, interpret, and react to social situations (which, in turn, are rooted in social knowledge structures, the “data base”), victimization experiences shape future expectations regarding other people’s trustworthiness. These expectations become increasingly stable via self- and environmental stabilization, and, especially, via person-environment “transactions.” Stabilized and generalized untrustworthiness expectations in conjunction with a strong need to trust make a person dispositionally sensitive to victimization—the “dependent variable” in our model (see Figure 1). Victim sensitivity, in turn, feeds back into the “data base;” that is, victim sensitivity shapes how people perceive, interpret, and react to similar situations containing similar cues (in the SeMI model, this is referred to as the “suspicious mindset;” cf. Gollwitzer and Rothmund, 2009; Gollwitzer et al., 2013).

We have also argued that late childhood and early adolescence may be a particularly critical age for the formation and stabilization of victim sensitivity, because both (a) the need to trust others—especially peers, friends, and partners—and (b) the likelihood of being confronted with instances of victimization are particularly high during this phase. To date, there are no empirical studies in which the stabilization of victim sensitivity in adolescence is systematically investigated. The only study that may be informative in this regard has been published by Bondü and Krahé (2014). These authors have shown that victim sensitivity can be reliably assessed and distinguished from other constructs by the age of 9. In this study, the predictive effect of victim sensitivity over and above other factors (e.g., rejection sensitivity) on aggressive behavior was examined in a large sample with ages ranging between 9 and 19 years. Victim sensitivity turned out to be the strongest predictor of various forms and functions of aggressive behavior. Interestingly, victim sensitivity was the only variable that increased as children grew older. This is in line with Flanagan and Stout’s (2010) finding that social trust declines during adolescence.

## How Does Victim Sensitivity Perpetuate Itself Across Social Situations?

After having discussed the “ontogenetic” development and stabilization of victim sensitivity across the life course, we will now turn to our second question: how do specific instances of victimization contribute to a stabilization of victim sensitivity across situations? This question addresses the “actual-genetic” stabilization of victim sensitivity. We will argue that this stabilization can be reasonably well explained by associative learning and avoidance learning processes. As outlined above, victim-sensitive individuals are not only characterized by a high need to trust but also by a stabilized and generalized negative expectation concerning others’ trustworthiness—probably due to experiences of victimization. These experiences are relevant for associative learning processes. According to the SeMI model, victim-sensitive individuals are particularly sensitive toward “cues” in their social environments that are associated with untrustworthiness (Gollwitzer et al., 2013). Being confronted with these cues evokes a “suspicious mindset” and makes preventive reactions, such as pre-emptive selfishness, more likely. Associative learning can explain why and how a sensitivity to “untrustworthiness cues” generalizes and, thus, stabilizes across situations.

### Associative Learning, Untrustworthiness Cues, and Trusting Behavior

Associative learning refers to the process by which associations between stimuli (including behavior) are learned. Associative learning encompasses classical, operant, and evaluative conditioning. In *classical conditioning* (or Pavlovian conditioning), a neutral stimulus is paired with an unconditioned stimulus (i.e., a reflex-evoking stimulus) until the neutral stimulus acquires the unconditioned stimulus’ capability to evoke the reflex; thus, a *stimulus-outcome association* is learned. In *operant conditioning*, a behavior is paired with a pleasant (reinforcement) or unpleasant (punishment) stimulus/action until the frequency of the behavior is changed; thus, a *behavior-outcome association* is learned. In *evaluative conditioning*, a neutral stimulus is paired with an affective stimulus until the neutral stimulus acquires the valence of the affective stimulus; thus, a *stimulus-stimulus association* is learned. In the following, we discuss three processes that are relevant for the generalization (and, thus, the stabilization) of victim sensitivity across situations.

#### Conditioned Stimuli

A first relevant assumption is that previously unconditioned stimuli that are associated with victimization become “conditioned.” After this association is learned, such stimuli function as “untrustworthiness cues” that activate a suspicious mindset among victim-sensitive individuals (Gollwitzer and Rothmund, 2009). Importantly, whereas some untrustworthiness cues are rather idiosyncratic (e.g., the first name of a perpetrator, the location at which a victimization took place), others are more universal: expressions of anger (e.g., angry facial expressions, aggression-related behavioral patterns, hostile verbal remarks) are arguably less idiosyncratic untrustworthiness cues as they are associated with perceived aggression and victimization in general. In one of our recent studies (Gollwitzer et al., 2012), participants rated the trustworthiness of targets whose emotional facial expression varied from happy to angry. Results showed that victim-sensitive persons were more distrustful of angry (and neutral) but not of happy faces. We suggest that associative learning can explain how certain stimuli, such as angry facial expressions or even behavioral cues (such as a colleague asking for a favor), become “untrustworthiness cues” for victim-sensitive individuals. However, it is important to keep in mind that victim sensitivity is assumed to entail a heightened responsiveness to *any* information that indicates untrustworthiness, irrespective of how that information was acquired. Thus, instances of *observed* non-cooperation (Gollwitzer et al., 2009) or the activation of culturally shared stereotypes (e.g., untrustworthy car salesman) might suffice to trigger a suspicious mindset in relevant contexts.

#### Exploitation as Punishment

Two other processes that are relevant for explaining the stabilization of victim sensitivity are operant conditioning (via punishment) and avoidance learning. Punishment occurs when cooperative behavior (e.g., telling a friend a secret, agreeing to switch shifts with a colleague) is followed by victimization (e.g., being betrayed, learning that one’s helpfulness was exploited). In line with operant conditioning, one might say that one’s willingness to trust others was “punished” and therefore becomes less likely to occur. Furthermore, behavior that reduces the likelihood of victimization becomes more frequent (via avoidance learning; see Avoidance Learning and the Stabilization of Victim Sensitivity).

#### Implicit Cognition

Finally, direct (and observed) experiences of victimization may not only influence people’s *explicit* evaluations of others’ trustworthiness (via propositional processes), but are also likely to affect people’s *implicit* evaluations of others (via associative processes). More precisely, victim-sensitive individuals might implicitly associate other people with untrustworthiness. According to the affective-propositional evaluation (APE) model (Gawronski and Bodenhausen, 2006), such negative implicit evaluations of others are particularly likely to drive explicit evaluations and behaviors in situations in which no inconsistent propositional information is considered (e.g., failing to realize that a different colleague requesting a favor has demonstrated her trustworthiness in the past), or in situations in which self-regulation resources are low (e.g., after having suppressed one’s bad mood at work for a while, see Hofmann et al., 2007). By default, victim-sensitive individuals’ evaluation of a new interaction partner can thus be understood as an affirmation of more general negative implicit expectations of others (Gilbert, 1991) unless contradictory trustworthiness cues are present. Taking victim-sensitive individuals’ implicit evaluations of others’ trustworthiness into account might be particularly important when it comes to changing their expectations of others’ (un)trustworthiness. Whereas the APE model describes a number of ways in which implicit associations can be influenced, research on evaluative conditioning suggests that affective reactions are highly resistant to extinction (De Houwer et al., 2001) and, thus, more difficult to alter than individuals’ explicit beliefs.

To sum up, we assume that associative learning plays a key role in the explanation of (a) victim-sensitive individuals’ heightened responsiveness toward certain untrustworthiness cues, (b) victim-sensitive individuals’ reduced trusting behavior, and (c) their implicit evaluations of other people’s trustworthiness (and accompanied affective reactions). Importantly, whereas probably all people have been victimized in their lives to some extent, we assume that victim-sensitive individuals not only have *more* of these aversive experiences, but also that they experience them more *intensely* due to their strong need to trust. More concretely, a high need to trust is likely associated with more attention and stronger negative emotions elicited by experiences of victimization (cf., Gollwitzer and Rothmund, 2011), thereby rendering these experiences psychologically more meaningful. Thus, a high need to trust exacerbates associative learning in victimization experiences because it increases the intensity of the unconditioned stimulus (Passey, 1948; Pearce and Hall, 1980)—especially if this stimulus occurs unpredictably and uncontrollably (see our discussion of critical life events in Section “When and How Does Victim Sensitivity Begin to Emerge and Stabilize?”; cf. Filipp and Aymanns, 2010).

### Avoidance Learning and the Stabilization of Victim Sensitivity

Avoidance learning is a basic learning principle that refers to a process of behavior modification by which an animal or human reduces exposure to an aversive stimulus through an avoidance response. In early studies on avoidance learning (e.g., Mowrer and Miller, 1942), animals learned that an aversive stimulus (e.g., electric shock) was preceded by a warning signal (e.g., a tone). The aversive stimulus could, however, be postponed with a certain response (e.g., change of location). Avoidant behavior demonstrated in these studies could not be explained by a purely behavioristic stimulus-response pattern because the avoidant response occurred without direct reinforcement (it was in fact driven by the *non-occurrence* of an aversive stimulus). Consequently, avoidance learning was explained as a combination of two factors: classical and operant conditioning (Mowrer, 1947). First, due to its pairing with the unconditioned stimulus (e.g., the electric shock), the former neutral stimulus (e.g., the tone) becomes a conditioned stimulus (i.e., classical conditioning). Importantly, the conditioned stimulus is assumed to elicit fear when it occurs. Second, when the organism then happens to perform the avoidance response in the presence of the conditioned stimulus and thus prevents the occurrence of the unconditioned stimulus, the fear elicited by the conditioned stimulus is reduced. This, in turn, reinforces the avoidance response (i.e., operant conditioning). Thus, avoidance learning is assumed to be driven and maintained by feelings of fear. However, cognitive aspects such as expectations are likely to be involved in human avoidance learning as well (see Rescorla and Wagner, 1972; Lovibond, 2006; Declercq et al., 2008).

In clinical psychology, avoidance learning is considered a crucial factor for the maintenance of anxiety disorders; it refers to the process by which individuals reduce their exposure to a phobic stimulus through avoidant behavior (Bouton et al., 2001; Mineka and Zinbarg, 2006). Trying to avoid an aversive stimulus deprives the individual of positive learning experiences in which the conditioned stimulus might *not* be followed by the aversive stimulus. Thus, avoidant behavior is strongly self-reinforcing.

Associative learning and avoidance learning are likely to play a central role for the stabilization of victim sensitivity across situations. As described earlier, operant conditioning can explain how behavior related to victimization—such as cooperation and trusting others (e.g., doing a colleague a favor)—becomes less frequent when followed by victimization (i.e., “punishment”). Consistent with this notion, highly victim-sensitive individuals have been found to withdraw their cooperation in a trust game after experiencing victimization in an entirely different context, such as a virtual world (e.g., Rothmund et al., 2011). Furthermore, classical conditioning can explain how stimuli that indicate victimization (such as angry facial expressions) are learned and become untrustworthiness cues. Drawing on avoidance learning, we assume that due to their connection with victimization, untrustworthiness cues may elicit fear as a conditioned response.

### Uncooperative Behavior as a Defense Reaction

Confrontations with “untrustworthiness cues” signal a threat to a particular need, the need to trust (see The Need to Trust). As described in the previous section, one way to cope with this threat would be to avoid the threat. Victim-sensitive individuals should tend to avoid situations in which they might fall prey to the egoistic intentions of others and instead prefer situations in which exploitation is unlikely. For instance, victim-sensitive individuals can be expected to prefer individual (i.e., independent) over cooperative (i.e., interdependent) work situations and situations in which free-riding is rigorously punished over situations in which free-riding is unlikely to be detected. Of course, these situational preferences also have an impact on the quantity and quality of their friendships and, especially, the extent to which a close relationship remains stable and satisfactory for both partners (cf. Gerlach et al., 2012).

However, research shows that victim sensitivity is not exclusively related to avoidance-oriented behaviors; victim-sensitive individuals show typical “approach-oriented” behaviors as well: whenever untrustworthiness cues are present, victim-sensitive individuals tend to behave uncooperatively in social dilemma situations (Fetchenhauer and Huang, 2004; Gollwitzer et al., 2009; Rothmund et al., 2011), even at the cost of their own benefit. Notably, victim-sensitive persons are not more egoistic *per se*; rather, they tend to be more hostile when faced with injustice. For instance, when given the opportunity to punish a defector or to compensate a victim in a third-party intervention game, victim-sensitive individuals prefer punishing the offender over compensating the victim, even if punishment is costly for them (Lotz et al., 2011).

The General Process Model of Threat and Defense (Jonas et al., 2014) provides a helpful and informative theoretical framework for explaining why and when avoidance-oriented behaviors turn into approach-oriented ones. This model posits that being confronted with threat (of any kind) first activates the behavioral inhibition system (including anxious arousal and attentional vigilance toward fear-eliciting cues) and facilitates avoidance-oriented defense reactions. Since a state of avoidance is perceived as inherently unpleasant, avoidance-oriented behaviors eventually turn into approach-oriented behaviors. These approach-oriented behaviors can be more or less concrete (e.g., seeking stimulation or social affiliation; attacking the source of the threat) vs. abstract (e.g., increased adherence to personal and moral values; endorsing punitive systems).

Regarding victim sensitivity, it is reasonable to assume that, when confronted with untrustworthiness cues, victim-sensitive individuals initially show avoidance-oriented reactions such as an increased attentional vigilance toward untrustworthiness. Prior research has shown that, even in the absence of an untrustworthiness prime, victim-sensitive persons show a greater attentional vigilance toward justice- and injustice-related semantic concepts (Baumert et al., 2012), and more recent research shows that, in the presence of an untrustworthiness prime (i.e., a victimization experience), victim-sensitive individuals are more likely to associate ambiguous social situations with injustice (Maltese et al., 2014). Especially the latter finding is in line with the notion that victim-sensitive individuals show avoidance-oriented reactions after being confronted with untrustworthiness cues. Avoidance, however, may eventually transform into approach, such as hostility, uncooperativeness, and recklessness. In other words, avoidance- and approach-related behaviors can be positively related to each other.

According to the General Process Model of Threat and Defense (Jonas et al., 2014), hostile, uncooperative, aggressive, and cynical behavioral reactions toward experienced or anticipated victimization can be regarded approach-related reactions that aim to defend or satisfy a certain need: for victim-sensitive persons, it is the need to trust. Such distal defense reactions tend to reinforce themselves, as we have discussed before. Uncooperativeness and selfishness as “pre-emptive” reactions to anticipated victimization therefore stabilize over time. Notably, such selfishness may backfire: Other people may take the “pre-emptive” selfishness displayed by victim-sensitive individuals as a cue for the fact that these individuals cannot be trusted, and behave uncooperatively in return. This, in turn, confirms what victim-sensitive individuals had expected. The pre-emptive selfishness that victim-sensitive persons are likely to display in social interdependence situations and the fear of exploitation that triggered this hostility both create a self-reinforcing system; a self-fulfilling prophecy.

Taken together, experiences of victimization increase avoidance-related (e.g., attentional vigilance toward untrustworthiness cues) *and* approach-related behaviors (e.g., pre-emptive selfishness). Whereas direct experiences of victimization are the starting ground for these processes to unfold, the nature of the behavioral reactions toward them contributes to the stabilization of victim sensitivity across situations. Because avoiding social exchange and social dilemma situations deprives individuals of contrary learning opportunities (e.g., changing shifts with a colleague who does you a favor in return) and because pre-emptive selfishness as an approach-oriented response will generate non-cooperation in response (e.g., loafing in a joint task), these behaviors eventually reinforce negative expectations concerning others’ trustworthiness.

### Conclusion

**FIGURE 2:**
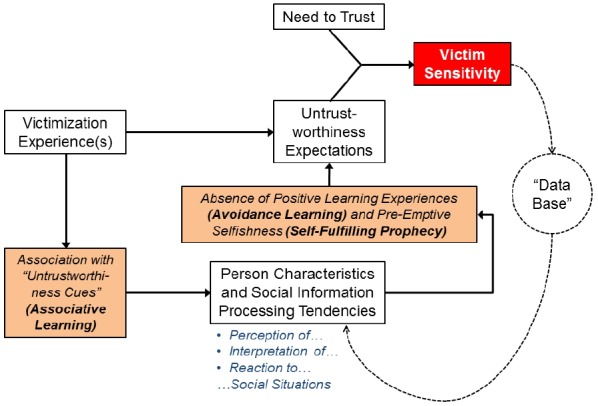
**Theoretical model explaining the “actual-genetic” stabilization of victim sensitivity across situations**.

In Section “How Does Victim Sensitivity Perpetuate Itself Across Social Situations?” of this article, we focused on the role of general learning mechanisms for the formation and stabilization of victim-sensitive individuals’ biased responses to untrustworthiness cues as well as their non-cooperative behavior. The model that results from these arguments is displayed in Figure 2. Associative learning can explain how victimization experiences result in (a) a generalization of untrustworthiness cues (via associative learning), (b) decreasing levels of trusting behavior (via operant conditioning due to punishment), and (c) the stabilization of negative implicit trustworthiness expectations. In addition, avoidance learning and self-fulfilling prophecies create a self-reinforcing cycle which stabilizes generalized untrustworthiness expectations as well as low trusting behavior both via avoidant and pre-emptively selfish or hostile behavior.

Notably, some of the processes we discussed with regard to the “actual-genetic” stabilization of victim sensitivity in the present Section can be meaningfully related to the four person-environment transactions that we discussed with regard to the “ontogenetic” stabilization of victim sensitivity in Section “When and How Does Victim Sensitivity Begin to Emerge and Stabilize?”. For instance, by selectively seeking social environments that reinforce their untrustworthiness expectations (“selective transactions” according to Caspi and Roberts, 1999, 2001), victim-sensitive individuals never challenge these expectations—which resembles an instance of avoidance learning. And self-fulfilling prophecies, as we defined them here, resembles what Caspi and Roberts (1999, 2001) referred to as evocative transactions: victim-sensitive individuals behave in ways that indirectly validate their beliefs that others are untrustworthy.

## Summary and Outlook

In this article, we developed a theoretical framework (or, rather, two theoretical frameworks) that aim at explaining how and why victim sensitivity emerges and stabilizes. Notably, victim sensitivity is not only a risk factor for antisocial behaviors in various kinds of social encounters (e.g., Gerlach et al., 2012; Gollwitzer et al., 2013), but also for a number of behavioral problems during adolescence, such as aggressiveness (Bondü and Krahé, 2014), anxious and angry rejection sensitivity and conduct problems (Bondü and Elsner, 2015) as well as symptoms related to attention deficit/hyperactivity disorders (Schäfer and Kraneburg, 2012; Bondü and Esser, 2015).

In Section “When and How Does Victim Sensitivity Begin to Emerge and Stabilize?”, we borrowed concepts from developmental psychology, research on coping with critical life events, and life-span personality psychology to derive a model that explains the “ontogenetic” stabilization of victim sensitivity during the life span. Victimization experiences and social information processes that describe how a person copes with these experiences are assumed to play a major role for the stabilization of victim sensitivity—more precisely, for the tendency to expect other people to be untrustworthy. From this model, which is depicted in Figure 1, testable hypotheses can be derived.

*First*, we assume that victimization experiences during late childhood and early adolescence increase a person’s victim sensitivity especially when these experiences are (a) self-relevant, (b) imply an obstruction of relevant personal goals, (c) are unpredictable, and (d) uncontrollable—in other words, when these experiences fulfill the criteria of “critical” life events. Examples for such events could be experiences of being bullied, cyber-mobbed, or socially excluded by significant peers. *Second*, we hypothesize that victim-sensitive individuals actively contribute to a stabilization of this trait by reacting consistently to potential victimization situations (“reactive transactions”). More precisely, we assume that victim sensitivity provides people with a set of cognitive schemas (e.g., attributional styles regarding other people’s untrustworthiness) and behavioral scripts (e.g., behaving uncooperatively) that bias their information processing in specific situations—situations that are marked by social interdependence and uncertainty regarding other people’s intentions and behaviors (i.e., social dilemma situations). A *third* hypothesis that can be deduced from our framework is that victim-sensitive individuals actively select environments (e.g., peers, friends, partners, etc.) that fit their own attitudes and worldviews (“selective transactions”). Such a fit between personality and the social environment reinforces victim sensitivity and stabilizes it over time. All of these hypotheses can be tested in carefully designed cohort—or, even more preferably, longitudinal—studies in which the variables that are assumed to predict the formation and stabilization of victim sensitivity are either measured or experimentally manipulated. We believe that late childhood to mid-adolescence is a critical phase for the formation and stabilization of victim sensitivity. Thus, cohort studies should at least compare age groups ranging between 9 and 15 years (Bondü and Elsner, 2015).

In Section “How Does Victim Sensitivity Perpetuate Itself Across Social Situations?”, we borrowed concepts from research on associative learning and social cognition to explain why and how victim sensitivity perpetuates across social situations. Associative learning can explain how neutral stimuli can become “untrustworthiness cues” for victim-sensitive persons, and avoidance learning can explain why social expectations regarding the untrustworthiness of other people reinforce themselves. In addition, approach-oriented behavior such as “pre-emptive” hostility and selfishness, which may be regarded a distal defense to threats to the “need to trust,” create a vicious cycle or a self-fulfilling prophecy: the degree of pre-emptive hostility displayed by victim-sensitive individuals in the face of untrustworthiness cues may lead their interaction partners to infer that cooperation is futile, which, in turn, reinforces the expectations held by victim-sensitive individuals. Again, a number of predictions follow from the framework we developed in Section “How Does Victim Sensitivity Perpetuate Itself Across Social Situations?” (see also Figure 2).

*First*, untrustworthiness cues are “stronger” unconditioned stimuli for people high (than for people low) in victim sensitivity. This hypothesis could be tested in an evaluative conditioning study featuring untrustworthiness and trustworthiness cues as well as neutral stimuli. In such a design, participants’ victim sensitivity should predict the change of liking toward neutral stimuli that were paired with untrustworthiness cues (but not with trustworthiness-related or neutral cues). *Second*, victim-sensitive individuals should harbor negative implicit evaluations of others’ trustworthiness due to associative learning. Using a single-target Implicit Association Test, it could be investigated whether victim-sensitive individuals associate “others” more readily with untrustworthiness relative to trustworthiness. More importantly, the influence of participants’ implicit untrustworthiness expectations on behavior (i.e., cooperation) should be examined *vis-à-vis* their explicit untrustworthiness expectations (i.e., victim sensitivity) in different situations (e.g., under ego depletion; in the presence vs. absence of trustworthiness information). *Third*, drawing on avoidance learning as well as the General Process Model of Threat and Defense (Jonas et al., 2014), we assume that in potentially exploitative situations, victim-sensitive individuals will first show avoidance-related reactions (e.g., a higher attentional vigilance to untrustworthiness cues), which eventually transform into approach-related reactions (e.g., “pre-emptive” selfishness). *Fourth*, victim-sensitive individuals contribute to the confirmation of their expectations and create cycles of non-cooperation through their own behavior (self-fulfilling prophecy or “evocative transactions”). This hypothesis could be tested in a repeated public goods game in which players have to decide how much to contribute to a common good (and do so iteratively for a number of rounds). In such a paradigm, we would expect, for instance, that one highly victim-sensitive individual eventually reduces the other players’ willingness to contribute, which confirms this individual’s *a priori* expectation: that other people are untrustworthy and harbor mean intentions.

To sum up, research on victim sensitivity, and on justice sensitivity in general, has gained momentum in various areas during recent years, and most of what we know about this trait so far is that it is a double-edged sword: in a way, it represents a true concern for justice and trust, but this concern leads to maladaptive behavioral decisions when social situations become uncertain. Thus, understanding how such a trait that is correlated with so many problematic behavioral tendencies emerges and stabilizes is therefore of vital importance, not only from a theoretical, but also an applied psychological perspective.

### Conflict of Interest Statement

The authors declare that the research was conducted in the absence of any commercial or financial relationships that could be construed as a potential conflict of interest.

## Footnotes

1 Genome-related stabilization effects will not be further discussed in this text since this would go beyond the scope of this article. It should be noted, however, that twin studies have demonstrated that a considerable amount of the variance in social trust is accounted for by an additive genetic component (e.g., Oskarsson et al., 2012).
